# Biomimetic Aspects of Restorative Dentistry Biomaterials

**DOI:** 10.3390/biomimetics5030034

**Published:** 2020-07-15

**Authors:** Muhammad Sohail Zafar, Faiza Amin, Muhmmad Amber Fareed, Hani Ghabbani, Samiya Riaz, Zohaib Khurshid, Naresh Kumar

**Affiliations:** 1 Department of Restorative Dentistry, College of Dentistry, Taibah University, Al Madinah, Al Munawwarah 41311, Saudi Arabia; hghabbani@taibahu.edu.sa; 2 Department of Dental Materials, Islamic International Dental College, Riphah International University, Islamabad 44000, Pakistan; 3 Science of Dental Materials Department, Dow Dental College, Dow University of Health Sciences, Karachi 74200, Pakistan; faiza.ameen@duhs.edu.pk; 4 Adult Restorative Dentistry, Dental Biomaterials and Prosthodontics Oman Dental College, Muscat 116, Sultanate of Oman; mafareed@staff.omandentalcollege.org; 5 School of Dental Sciences, Universiti Sains Malaysia Health Campus, Kubang Kerian 16150, Kelantan, Malaysia; samiya80@student.usm.my; 6 Department of Prosthodontics and Dental Implantology, College of Dentistry, King Faisal University, Al-Ahsa 31982, Saudia Arabia; drzohaibkhurshid@gmail.com; 7 Department of Science of Dental Materials, Dow University of Health Sciences, Karachi 74200, Pakistan; kumar.naresh@duhs.edu.pk

**Keywords:** dental biomaterials, endodontics, restorative dentistry, regenerative medicine, tissue engineering

## Abstract

Biomimetic has emerged as a multi-disciplinary science in several biomedical subjects in recent decades, including biomaterials and dentistry. In restorative dentistry, biomimetic approaches have been applied for a range of applications, such as restoring tooth defects using bioinspired peptides to achieve remineralization, bioactive and biomimetic biomaterials, and tissue engineering for regeneration. Advancements in the modern adhesive restorative materials, understanding of biomaterial–tissue interaction at the nano and microscale further enhanced the restorative materials’ properties (such as color, morphology, and strength) to mimic natural teeth. In addition, the tissue-engineering approaches resulted in regeneration of lost or damaged dental tissues mimicking their natural counterpart. The aim of the present article is to review various biomimetic approaches used to replace lost or damaged dental tissues using restorative biomaterials and tissue-engineering techniques. In addition, tooth structure, and various biomimetic properties of dental restorative materials and tissue-engineering scaffold materials, are discussed.

## 1. Introduction

The phrase “biomimetic” was coined by biophysicist/biomedical engineer Otto Schmitt in the 1950s [1,2] and refers to the study of multi-disciplinary mechanisms and biologically produced materials to design novel products to mimic nature [1,3]. Biomimetic is derived from Latin word “bio” meaning life, and “mimetic” is related to the imitation or mimicking biochemical process with inspiration from nature. Several biomimetic synonyms are used in the literature, for example, bionics, bioinspiration, biogenesis, biomimicry, biomimicking, and biomimetism. Novel approaches have produced hierarchal structures by accumulating inorganic ions in a coordinated manner along with organic protein molecules analogous to biomineralization [4]. Therefore, the understanding of emerging biomimetics approaches has involved the conception of multiple ideas from biology, chemistry, materials science, and bioengineering. In addition, numerous innovations of materials at nanoscale have accentuated a major push in the fabrication of biomimetic materials using nanotechnology [2]. It is encouraging to witness the emergence of biomimetic courses in educational sectors of robotics engineering, interdisciplinary teaching, biomaterials, and industrial design for undergraduate as well as postgraduate students [5,6]. In this regard, Cleymand et al. discussed the concepts and real examples of biomimetic principles and tools for the development of new materials, new improved design and fabrication strategies, and innovation methodologies used for students in a “biomimicry” course [5]. 

Biomimetic approaches were extensively explored across various disciplines including dentistry. Contemporary dentistry involves the minimal invasive dental management of defective or diseased tissue with bioinspired materials to achieve remineralization. The instrumental role of fluoride to control incidence and in prevention of dental caries has been widely reported in the literature for over a quarter of a century [7,8,9]. More recently, a variety of bioactive formulations such as micro- and nano-hydroxyapatite (HA), tricalcium phosphate, mineral trioxide, casein-phosphate, and bioactive glasses have been advocated due to their excellent biocompatibility, biomimicry, bioactivity, and remineralization potentials [10,11]. 

In clinical dentistry, biomimetics refers to the repair of affected dentition mimicking the characteristics of a natural tooth in terms of appearance, biomechanical, and functional competences [12]. For example, adhesive restorative materials have demonstrated tooth morphology and esthetics mimicking natural teeth [13]. Similarly, biomimetic dental implant coatings of calcium phosphate (CaP) and HA have been investigated to improve osseointegration of dental implants to achieve therapeutic benefits [14,15,16]. In addition, tissue-engineering approaches have reported promising results in regeneration of oral tissues [17,18]. Biomimetic endodontic regeneration includes the formation of dentin barrier by pulp-capping agents, root formation during apexogenesis and apexification, apical healing by root-end fillings, and pulp regeneration by cell-homing strategies [19,20,21]. The aim of the present article is to review various biomimetic approaches used to replace lost or damaged dental tissues by means of restorative biomaterials and tissue-engineering techniques. In addition, tooth structure, and various biomimetic properties of dental restorative materials and tissue-engineering scaffold materials, are discussed. 

## 2. Dental Hard Tissues

**Figure 1:**
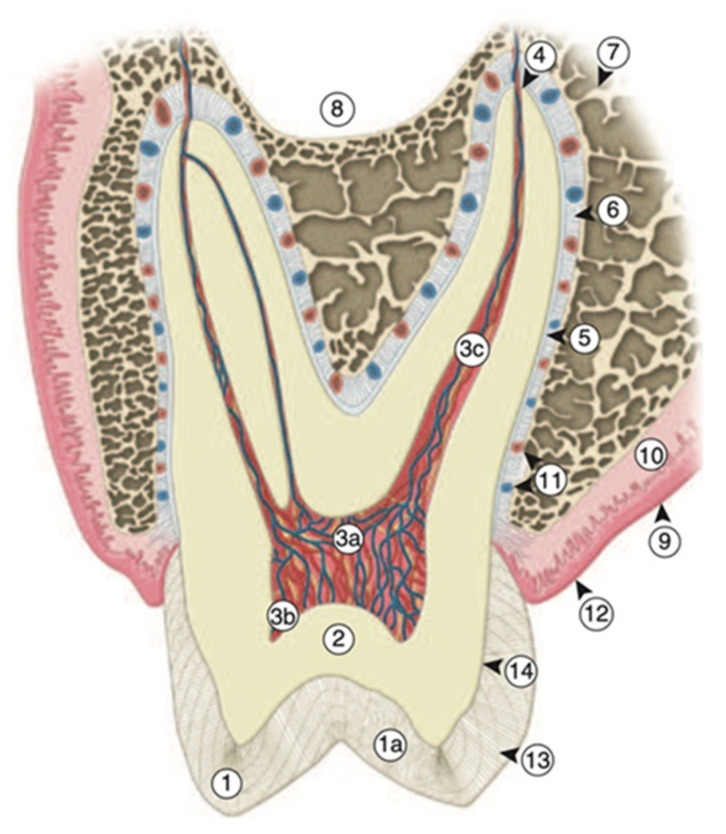
A cross-section of a tooth showing structural features of natural dental tissues; (**1**) Enamel; (**1a**), gnarled enamel; (**2**) dentin; (**3a**) pulp chamber; (**3b**) pulp horn; (**3c**) pulp canal; (**4**) apical foramen; (**5**) cementum; (**6**) periodontal ligament; (**7**) alveolar bone; (**8**) maxillary sinus; (**9**) mucosa; (**10**) submucosa; (**11**) blood vessels; (**12**) gingiva; (**13**) lines of Retzius; (**14**) DEJ. (Reproduced from [22] with permission from the publisher).

Natural teeth are always considered to be a reference while employing biomimetic approaches to restore diseased or fractured dental tissues. Human teeth have a complex structure with an inner core of highly vascular, soft, and delicate pulp surrounded by the highly mineralized enamel and dentin tissues (Figure 1). In the dynamic oral environment, the mechanism of remineralization and demineralization simultaneously coexist throughout the life of a tooth. Therefore, brief anatomical characteristics of structural components of human teeth, including enamel, dentin–pulp complex (DPC), and cementum are described to understand biomimetic strategies. 

### 2.1. Enamel 

Enamel is a formed by the highly mineralized crystalline lattice structure of HA (90–92% by volume), organic matrix proteins (1–2% by volume), and water (4–12% by volume). The thickness of enamel varies in different areas of anatomical sites in various teeth; for example, it is thinner at the cementoenamel junction (CEJ) compared to the occlusal/incisal surface [22]. The average enamel thickness varies from 2 mm at the incisal edge, 2.3–2.5 mm at the premolar cusp, and 2.5–3 mm at the molar cusps. It is interesting how food movements take place when functional cusps occlude on enamel inclines of opposite teeth to move the bolus to the facial and lingual surface of the teeth due to the strategic placement of cusps opposing to grooves and fossae [23]. On the enamel surface, a fissure is the deep invagination in the grooved area whereas a pit is the non-coalesced enamel at the fossa’s deepest point. These pit and fissure areas are potential sites of biofilm accumulation, demineralization, and dental caries [24]. 

Amelogenesis refers to the development of enamel, which is initiated by ameloblast cells originating from the ectorderm embryonic germ cell layer. The microscopic structure of enamel is composed of enamel rods (enamel prisms), rod sheaths, and inter-rod material. The approximate number of enamel rods varies from 5 to 12 million between mandibular incisors and maxillary molars, respectively. The resulting variation during structural and mineral formation of enamel rods is known as incremental striae of Retzius (growth rings) which are formed during amelogenesis (Figure 1). Enamel rods are generally aligned at a right angle (<90°) at the junction of enamel with dentin, i.e., dentino-enamel junction (DEJ), except at the cervical region of a tooth surface where rods are positioned in apical direction. There is also a 30 µm-thick prismless enamel around the cervical area which has a more heavily mineralized layer of enamel [25]. 

Although enamel has a hard, dense crystalline structure, it is still permeable to certain ions and molecules which may pass through rod sheaths due to crack formation and hypo-mineralized enamel structure [25]. The organic contents of enamel and water have a significant role in transporting ions through inter-crystalline enamel rod spaces [22]. However, due to enamel maturation with age, this permeability decreases with time. Enamel is brittle in nature and has a higher elastic modulus and compressive strength compared to more resilient and flexible dentin. Thus, it is important for the enamel rods to be supported by dentin for its strength as the ability of enamel to resist masticatory forces largely depends on the dentin through DEJ. Although enamel is the one of the hardest structures (hardness is lowest at DEJ), it solubilizes in the acidic environment due to bacterial attack in the oral cavity. It is well established that the enamel solubility decreases whereas hardness is increased with the adsorption of fluoride ions on the enamel surface. Fluoride has a significant role in reducing tooth decay by enhancing remineralization [7,8]. In addition, fluoride influences the chemical and physical properties of enamel, by altering the rate of demineralization, improving remineralization, and preserving apatite structures.

### 2.2. Dentin–Pulp Complex

Both dentin and pulp originate from the mesodermal layer of dental papilla of a tooth bud. Dentinogenesis or dentin formation is initiated by odontoblast cells, which are the part of both dentin and pulp tissue. Dentin is composed of inorganic (50% by volume) and organic material (30% by volume; 90% of which is type 1 collagen and 10% non-collagenous proteins). The cell bodies of odontoblasts are present in the pulp cavity while their Tomes fibers (long cytoplasmic cell process) reach the dentinal tubules which run throughout the dentin. Sometimes, the odontoblastic process (enamel spindles) reaches the enamel after crossing the DEJ. Dentinal tubules have dentinal fluid which helps in the process of mineralization [26]. Dentin covers most of the tooth structure and it is externally covered by enamel and cementum. Odontoblasts form the most recent dentin on the pulpal surface for the generation of an extracellular matrix, which is mineralized. The following are the different forms of dentin: (a) predentin (unmineralized dentin); (b) primary dentin (initial form of the tooth);(c) reparative dentin (irregularly formed in response to injury); (d) secondary dentin (regular circumferential dentin form after eruption); and (e) peritubular dentin (walls of the dentinal tubules) [7,8]. The sensitivity of dentin to thermal, chemical, physical, bacterial, and traumatic stimuli are detected via dentinal tubule fluid, i.e., hydrodynamic theory of stimulus detection because of rapid tubular fluid movements. Dentin is flexible, less mineralized, and softer than enamel but harder and more mineralized than cementum. The flexibility of dentin helps to provide a good support to brittle and non-resilient enamel [26]. 

Dentin and pulp are considered to be a single tissue unit, i.e., DPC. Pulp tissue is enclosed by dentin and lined by odontoblasts at the periphery of pulp tissue. Therefore, the integrated dynamics of DPC impact not only the quality and quantity of dentin but also the pulpal component. A cell-poor zone is present adjacent to the odontoblast layer in the coronal pulp but a cell-rich zone is present in the sub-odontoblastic area [25,27]. Moreover, the central pulpal mass containing blood vessels, nerves, connective tissue, and fibroblasts is known as pulp proper (Figure 1). The most common characteristic pulp cells are odontoblasts, odontoblast process, fibroblasts, macrophages, lymphocytes, mast cells, and dendritic cells [25,27]. DPC has an excellent inherent repair and healing potential due to the presence of macrophages, proliferation of fibroblast, and collagen [25]. Some of the physiological functions of dental pulp are presented in Table 1.

### 2.3. Cementum

The anatomical structure of a tooth root is covered with as thin layer of avascular cementum formed by cementoblasts that are developed from mesenchymal cells of dental follicle. Cementum is composed of inorganic HA (45–55% by weight), organic matrix in the form of collagen and proteins (50–55% by weight) and, water [28]. Sharpey fibers are the periodontal collagen ligament, which are embedded in the cementum to attach the tooth to the bone. Cementum formation and deposition continues throughout the life of the teeth to keep the tooth attachment intact with the bone. Acellular cementum (without cementoblasts) is present in the coronal portion but cellular cementum is associated with the apical half of the root structure. Generally, the attachment of cementum with dentin is very durable; however, at CEJ in a few cases (10%) cementum does not meet with enamel, which may lead to sensitivity [25]. Interestingly, cementum has the potential to repair and regenerate and remain un-resorbed under the physiological occlusal forces [22]. 

## 3. Biomimetic Approaches for Dental Restorative Biomaterials 

From restorative dentistry perspectives, the biomimetic concept is highly relevant and mainly aims to process restorative materials in a way that matches the natural processing mechanisms of the oral environment. The secondary goal is to develop restorative materials that can mimic or restore the biomechanics of the natural tooth. The applicability of biomimetics has been greatly considered at molecular levels to augment the wound healing and soft and hard-tissue regeneration [29,30]. At a macrostructural level, the biomechanical, structural, and aesthetic integrity of teeth can be achieved by various biomimetic restorative materials. For this purpose, materials scientists would ideally consider natural teeth as a reference during the development of dental restorative materials. Accordingly, the tooth hard-tissue structures and associated characteristics are discussed in Section 2 and compared comprehensively with the characteristics of various restorative dental materials. 

The applicability of biomimetic principles can elicit innovations in restorative dentistry for tooth conservation and preservation. While restoring the damaged part of teeth, factors such as hues, shades, intra-coronal anatomy, mechanics, and position of teeth in the arch should be considered to respect the biomimetic principles [31]. Resin dental composites (RDCs), dental ceramics, and glass–ionomer cements (GICs) are commonly used to restore such features depending on the extent of damage and aesthetic requirement. Most of the dental ceramics and hybrid RDCs have potential to mimic the enamel and dentin, respectively. However, it has been suggested that moderate damage of teeth could be restored with RDCs [32]. For the RDC restorations, minimal preparation of teeth is required, which in turn can reduce the likelihood of pulpal involvement and tooth fracture. Moreover, RDCs have a potential to strengthen the remaining tooth structure when placed in tooth defects with low configuration factor [33]. In case of severe tooth damage, for instance wear or fractured teeth, bonded porcelain restorations are advocated [32]. Alumina and HA are the commonly used ceramics in dentistry. The former exhibits a good fracture resistance, wear behavior and higher compressive strength. However, HA is a main component of the teeth and bone; therefore, it is likely to achieve biomimetic characteristics in restorations. GICs are bactericidal as they release fluorides and have potential to stimulate sclerotic dentin. Moreover, these cements have characteristics comparable to dentin, thus fulfilling the concept of biomimetics [34]. GICs are used as restorative materials in deep class I or II cavities in pedodontics and for restoration of class V cavities. GICs are not generally recommended in load-bearing posterior dentition due to poor tensile strength.

### 3.1. Biomimetic Mechanical Perspective of Restorative Materials

In terms of mechanical perspective, elastic modulus [35,36,37,38,39,40,41] and surface hardness [42,43,44,45,46,47] of restorative materials are widely evaluated to predict the clinical performance. Elastic modulus (EM) is considered to be an intrinsic characteristic of materials and it gives a clear picture about the stiffness of materials. Ideally, the EM intrinsic characteristic of dental restorative materials should be harmonized with tooth hard tissues to facilitate uniform sharing of stresses in the region of tooth-restoration interface during the functional masticatory load. The gross discrepancy of EM across the tooth-restoration interface may enhance the probability of fracture of remaining tooth structure. In addition, tooth-restoration bonding may fail leading to microleakage and secondary caries [48,49]. The EM of dentin and enamel has been reported as 14–38 GPa and 72–125 GPa respectively [50,51] (Table 2). Therefore, an ideal dental restoration can be produced using a combination of two different dental restorative materials with the EM closely matching to the EM of enamel and dentin. In clinical practice, the direct restorative materials include RDCs and GICs that are filled in teeth on chairside to restore the lost tooth tissues. However, scientific literature suggests that some of the RDCs are meeting the EM values of dentin whereas, EM of GICs is far lower than the EM values of dentin and enamel (Table 2). Therefore, in the context of mismatched EM between enamel and the direct restorative materials, more stresses may be transferred to teeth which may lead to either tooth damage or failure of restoration. 

Despite the aforementioned mismatching of EM between tooth enamel and direct restorative materials, an acceptable survival rate up to 12 years of associated restorations have been reported in the retrospective clinical studies [52,53]. Consequently, fundamental concepts of load-sharing between teeth and direct restorative materials may be questioned. It is logical to state that success of restorative materials during clinical performance is not necessarily due to the superior mechanical properties. There may be other associated factors such as underneath adhesives applied prior to restoration. These adhesives exhibit flexibility thus are capable of absorbing the energy under the masticatory forces due to their resilience and eventually prevent failure of the restoration [54]. 

GICs exhibit lower EM compared to enamel, dentin, and RDCs. Accordingly, a longer clinical performance of GICs restoration is unlikely for the restoration of load-bearing areas due to weaker mechanical properties such as brittleness and surface wear, a high porosity in the mix and poor surface polish as reported in the literature [55,56]. Nevertheless, useful features of GICs for instance, their ability to release fluoride [8,57], anti-cariogenic properties, and chemical bonding with teeth are well evident [58,59,60]; however, such features are not adequate to make GICs suitable candidates for load-bearing restorations. Therefore, GICs are mainly employed as a cavity liner, luting agents for cementation of crowns and bridges, and small cavities especially in deciduous dentition. Among modern indirect restorative materials, the properties of dental ceramics notably elastic modulus, hardness, and thermal expansion are comparable with enamel. Therefore, in modern restorative dentistry, ceramic veneers can be preferably used for the damaged anterior dentition due to probable uniform distribution of the stresses across the tooth-restoration interface. Apart from the fulfilment of mechanistic considerations, ceramic laminates are useful candidates from the aesthetic perspectives [54].

In addition to the intrinsic properties of restorative dental materials, the surface characteristics are also relevant in the real clinical environment. Consequently, surface hardness (SH) of the restorative materials is determined so as to find their resistance to permanent surface indentation, which indirectly predicts the abrasion resistance and polishing ability of materials during their service in the oral environment [61]. Ideally, the SH of restorative materials should closely match to the hardness of enamel since surfaces of restorations are directly exposed to masticatory forces and moist atmosphere such as the tooth enamel. Therefore, restorative materials with lower SH are more susceptible to abrasion resulting in surface wear, porosity and eventual failure [62,63,64].

The tooth enamel is an extremely hard tissue. The SH of tooth enamel ranges from 2.23 to 7.18 GPa and dentin ranges from 0.71 to 0.92 GPa [50,51] (Table 3). The SH of direct restorative materials including RDCs and GICs are substantially lower compared to tooth enamel (Table 3) therefore are more susceptible to surface wear and failure. In contrast, SH of dental ceramics meets the limits of SH of natural tooth enamel. The rough surfaces of restorations encourage plaque accumulation leading to secondary caries and failure of restorations [65,66,67]. It is also pertinent to mention that the presence of porosities on surface of RDC restorations are likely to act as flaws in accordance with Griffith’s Law. These surface porosities may be considered to be nuclei, which may aid crack propagation and cause failure of restorations [68].

The aforementioned fundamental concepts of materials science clearly indicate that dental ceramics restorations are likely to perform better than direct RDCs and GICs restorations. However, the variables related to patients cannot be overlooked while predicting the longevity of restorations. Such variables include caries index, restoration size, location of tooth, rate or magnitude of masticatory forces, clinicians’ knowledge, and psychomotor skills [90]. Opdam et al. [53] assessed the 10-year survival rate of 1,955 resin dental composite restorations and reported a high success rate (82.2%). Dental students in Manitoba, Canada, placed 1695 two-surface RDCs restorations in premolars and after 12 years, the survival rate was calculated as 86%. Burke and Lucarotti [92] reported survival rate of GIC restorations up to 28% after 15 years of their placement. Layton [93] and Fradeani [94] have reported survival rate of 93% at 10 to 11 years and 94.4% at 12 years respectively for ceramics veneer restorations. In addition, an assessment of 1,588 ceramic inlay or inlay restorations revealed 97% survival at 10 years [95]. It is logical to assume that RDCs and dental ceramics are promising materials for dental restorations and closely mimic the mechanical and functional characteristics of natural teeth to a great extent. 

### 3.2. Aesthetics Perspective of Restorative Materials

**Figure 2:**
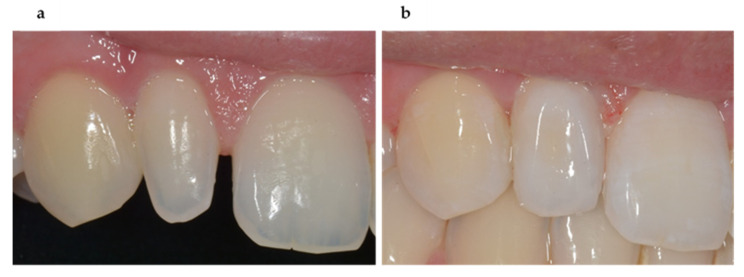
Restoration of peg-shaped lateral incisor using the direct restorative composites; (**a**), pre-operative image of lateral incisor showing peg shape (**b**), post-operative image showing the restoration of the defect matching morphology and color similar to the adjacent natural teeth.

Dental composites are presently the direct restorative materials that best accomplish the requirements of tooth conservation, outstanding aesthetics, and durability. RDCs are used for the various aesthetic problems namely discolorations, peg-shaped lateral incisors, diastema and misaligned teeth [96]. Modern RDCs kits comprise several shades and opacities for the corresponding translucency and shades of enamel and dentin that facilitate the clinician to provide highly aesthetic restorations to patients. Excellent aesthetic and optical properties enable clinicians to match color and morphology of RDCs restorations with tooth structure (Figure 2). Geengler et al. [97] reported that 93% of posterior RDC restorations exhibited a satisfactory color match to neighboring tooth structure after 10 years. Moreover, Wilder et al. [98] observed an excellent color matching of about 94% RDC restorations after 17 years of their placement. GIC restorative materials have poor aesthetic properties and are not usually not considered where esthetics is a main concern in anterior restorations [99].

**Figure 3:**
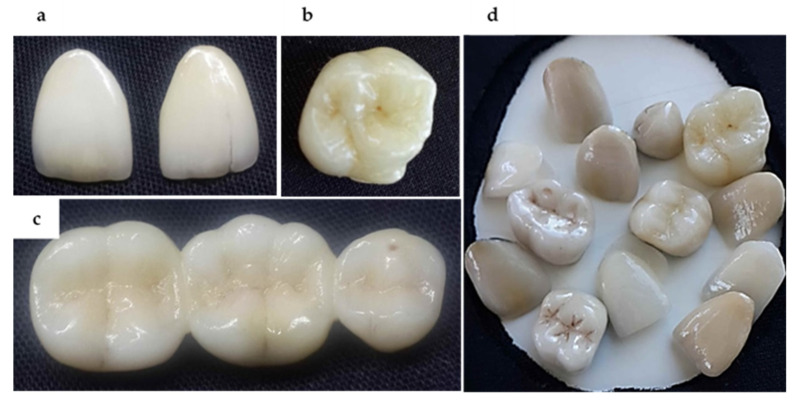
The indirect porcelain restorations demonstrating excellent esthetic properties; (**a**), anterior crown (**b**), posterior crown (**c**), bridge and (**d**), multiple indirect restorations showing variations in color, translucency and stains to match patients’ natural teeth.

In recent years, the porcelain veneer has gained extensive recognition as a primary restoration in esthetic dentistry. A variety of materials and techniques have been introduced since its development in the early 1980s. The long-term clinical studies reported the outstanding performance of the porcelain veneer restorations [94,100,101]. Owing to minimally invasive preparation and excellent esthetics, porcelain veneers have become the treatment of choice for restoration of the anterior dentition [102]. The indirect porcelain restorations (crowns, bridges, veneers) have demonstrated excellent esthetic properties (Figure 3) in terms of morphology, and optical properties (color, hue, translucency, and fluorescence) mimicking natural enamel. In addition, a variety of surface characteristics such as pits, fissures and stains can be introduced matching prosthesis to patient’s own dentition.

### 3.3. Biocompatibility of Dental Restorative Materials

Biocompatibility is an important biological property that is essentially required for restorative applications in the oral cavity. Ideally, a restorative dental material or its breakdown products should not incite any allergic or toxic reaction during the funtional performance. The majority of currently available biomimetic restorative materials are considered biocompatibile for their respective application. Although the cytotoxic reactions of chemical and light-cured RDCs following initial setting are reported in vitro, current RDCs exhibit minimal toxicity that is further decreased soon after their placement. In addition, in vivo findings are also in the agreement with in vitro results [103]. Similarly, a mild cytotoxicity of freshly mixed GICs has been reported in vitro studies; however, such toxicity reduces gradually [104]. Moreover, liquid component of GICs possess high molecular weight, hence, its diffusion in the underlying dentin may be reduced and as a result pulpal response may be minimized. The mild pulp reactions of the GICs have also been reported in the usage test and histological evaluation of these cements report minimal or absence of inflammatory response after 4 weeks’ time [105]. On contrary, dental porcelains are bioinert and highly biocompatible materials. The ceramic restorations are highly stable and insoluble and do not cause any harmful biological reactions. The scientific literature highlights relatively low biological adverse effects of dental ceramic in contrast to the direct restorative materials [106,107,108].

### 3.4. Biomimetic Mineralization of Enamel and Dentin: A Current Approach in Restorative Dentistry

There are certainly a wide range of variation between dental restoratives and tooth hard tissues in chemical and structural characteristics. The concept of biomimetic mineralization that mimics the natural mechanisms of tooth mineralization. is being considered to be a substitute to restorative approach The dental caries is a common disease of oral cavity worldwide and it occurs due to discrepancy between remineralization and demineralization phases of tooth hard tissues [109]. This perception of dental caries offers the possibility for remineralizing initial enamel caries. A variety of remineralizing approaches namely fluoride [110,111], surfactants [112], electrolytic deposition [113], hydrothermal methods [114], and hydrogen peroxide [115] have been introduced to restore initial enamel caries and inhibit further enamel demineralization Consequently, under physiological conditions, synthesis of enamel-like apatite structures through biomimetic approaches is considered to be a substitute restorative method. Encouraging outcomes with regard to enamel biomimetic mineralization by employing proteins and protein analogues, an agarose hydrogel model, bioactive materials or components, ethylenediaminetetraacetic acid and a glycerin-enriched gelatin technique have been reported [116].

In dentin, the mineral phase is affected by the initial carious lesions and it leads to exposure of the collagen fibers. Consequently, degradation of collagen fibrils and inferior mechanical properties of dentin are well evident [117]. In a systemic review, Cao et al. [118] have reported different methods for instance the application of non-collagenous proteins (NCP) analogues and bioactive materials for the biomimetic mineralization of dentin. The success of NCP analogues was observed in terms of their remineralization ability as intrafibrillar and interfibrillar remineralization of dentin collagen fibrils was well evident. Oral cavity has a complex dynamic and dynamic environment in which the restorative materials are exposed to a wide range of variations in terms of temperature, pH, microorganisms, and nutrients. The complex oral environment also varies vastly among individual depending on multiple factors such as age, ethnicity, and lifestyle. It is hard to mimic in vivo conditions during experimentation. It is pertinent to mention that the methods involved for the mineralization of tooth hard tissues are carried out under very low acidity or environments with high electric field, which are less likely to be translated to clinical applications. Thus, further investigations may enhance the potential for the biomimetic mineralization approach in restorative dentistry. 

## 4. Biomimetic Endodontics and Regenerative Aspects

There is a wide range of biomimetic applications in the endodontics including biomaterials (irrigation agents, intra-canal medicaments and cements) and tissue regeneration (dentin and pulp regeneration, revascularization). The irrigants, intra-canal medicaments and cements are reviewed here while the biomimetic endodontic tissue regeneration aspects are discussed in Section 5. 

### 4.1. Endodontic Irrigants

The main reason for the failure of conventional and regenerative endodontic procedures (REPs) includes persistent microbial infections [119] due residual microorganisms in the root canals. Therefore, disinfection of root canals using copious irrigation is essentially required to resolve the periapical infection without harming the dental tissues [120]. Disinfection by profuse irrigation agents eradicate infected and necrotic tissues from the root canals [121]. Accordingly, an endodontic irrigant should have antimicrobial property without harming the healthy tissues and stem cells [122,123,124]. In addition, irrigants’ ability to dissolve pulp remnants and necrotic tissues is necessary to facilitate removal of debris [125]. The necrotic pulp and the multiplying microorganisms may cause pulp necrosis, and spread of infection to localized facial tissues and bone resorption [126]. In terms of endodontic regeneration, the disinfection protocols must not harm the stem cells viability. Several irrigants are used for the irrigation of the root canals that alter the dentinal surface in the root canal by removing smear layer favoring regenerative procedures. Commonly used root canal irrigants are sodium hypochlorite (NaOCl), hydrogen peroxide (H_2_O_2_), chlorhexidine (CHX), ethylene diamine tetraacetic acid (EDTA), and saline. The chemical irrigants and intra-canal medicaments are used in combination to disinfect the root canals and eliminate inflammation favoring regenerative endodontic procedures. 

The effects of various irrigants on stem cell survival has been reported (Table 4). In an in vitro study, Trevino et al. isolated and expanded the apical papilla stem cells (SCAPs) from immature human third molars and evaluated the effects of different irrigation protocol on stem cells survival. The immunohistochemistry of cultures SCAPs after 21 days demonstrated that the dentin exposed to EDTA or NaOCl resulted in surviving SCAPs, whereas irrigation using CHX (2%) showed contrasting findings [127]. In addition, Martin et al. evaluated the effects NaOCl concentration on SCAPs survival and expression of dentin sialophosphoprotein (DSPP). The higher concentrations of NaOCl (>1.5%) demonstrated a negative effect on the survival and differentiation of SCAPs, while 1.5% concentration had a minimum effect on survival of SCAPs. Similarly, EDTA (17%) have a positive influence on SCAPs survival [128]. Galler et al. also investigated the effects of the different irrigants on the transforming growth factor-β1 (TGF-β1) release from the dentin [129]. In contrast, CHX irrigation demonstrated toxicity and negatively affected the survival rate of SCAPs. However, such effects can be controlled by reducing the irrigation time and neutralization using L-a-lecithin [130].

### 4.2. Intra-canal Medicaments

Intra-canal medicaments (ICM) are applied in the root canals to eliminate the residual microorganisms following cleaning and irrigating [131,132]. Several studies have investigated the effects of various ICM during endodontic treatment [133,134,135]. To assure the microbial elimination from the root canals, the supporting action of a disinfecting agent is mandatory. In addition, interappointment medicaments prevents regrowth of microorganisms in the empty pulp space [136]. The high concentration of ICM demonstrated harmful effects to SCAPs and dental pulp stem cells (DPSC) that may affect the outcome of endodontic regeneration [137]. Ruparel et al. investigated the effects of triple antibiotics paste (TAP), double antibiotics paste (DAP) and calcium hydroxide (Ca(OH)_2_) on in vitro SCAPs survival potential. SCAPs were cultured and exposed to various concentrations of TAP medicaments (ciprofloxacin, cefaclor and metronidazole), Augmentin and Ca(OH)_2_. In a concentration dependent fashion, all antibiotics remarkably reduced SCAP survival while the Ca(OH)_2_ was conducive to SCAP survival regardless of its concentration [137]. 

Similar findings reported by Althumairy et al. further validate that the dentin exposed to TAP or DAP lead to SCAPs viability. A significant increase in the survival and proliferation of SCAPs was reported in the Ca(OH)_2_ treated specimens [133]. Therefore, the type and concentration of ICM should have the antibacterial efficacy without causing any toxicity to the stem cells [133,137,138]. Considering the mixed nature of root canal microbiota, a combination of antibiotics is preferred. 

### 4.3. Biomimetic Endodontic Cements

#### 4.3.1. Calcium Hydroxide

Calcium hydroxide Ca(OH)_2_ is an alkaline (pH ~12.5) white odorless compound [139] and frequently used for several endodontic applications such as apexification and pulp-capping procedures [140]. Due to alkaline pH, Ca(OH)_2_ has antimicrobial properties [141]. Ca(OH)_2_ acts on the bacterial cells to denatures their proteins by disrupting the cytoplasmic membrane [142]. In addition, it induces remineralization, repair/regeneration of dentin and inhibits resorptive activity [140,143,144]. However, Ca(OH)_2_ application for a prolonged time during the apexification may weaken the root dentin by resorptive activity [145] and even fracture of the root [146]. The alkalinity of Ca(OH)_2_ is directly proportional to the availability of free hydroxyl ions (OH^−^) following its dissociation. Deep penetration of OH^−^ in to dentinal tubules raise the pH and solubilize organic matrix disrupting the organic-inorganic proportion of dentin [147]. 

In terms of regenerative endodontics, Ca(OH)_2_ at concentrations up to 500 mg/mL is conducive to survival of SCAPs. Researchers have demonstrated that adding Ca(OH)_2_ in lower concentration (1 mg/mL) to the culture medium increases the proliferation of SCAPs [133,148]. However, at higher concentrations, Ca(OH)_2_ may have toxic and unfavorable effect on SCAPs and required further investigations. The differentiation of stem cells in the peri-radicular resulted in completion of root [149]. Due to high alkaline pH and resorptive activity, the application of Ca(OH)_2_ for a prolonged time should be avoided. Another concern is the complete removal of Ca(OH)_2_ from the root canals due to the complex anatomy. Using copious irrigation (NaOCl and EDTA in combination) may leave remnants of Ca(OH)_2_ on to the retentive areas of root dentin [150]. To improve the removal of Ca(OH)_2_ from the root canals, manual instrumentation can be considered [151]. 

#### 4.3.2. Triple Antibiotic Paste

TAP is an ICM comprised of three antimicrobial agents including bacteriostatic (minocycline) and bactericidal (metronidazole, ciprofloxacin). These antibacterial agents target the complete eradication of microorganisms from the root canals and favoring the endodontic revascularization [40]. Ciprofloxacin belongs to floroquinones and is known for its good penetration [152], highly effective against anaerobes present in necrotic pulp [152,153] and safe as ICM even for pediatric patients in low concentration [154]. However, ciprofloxacin is not very effective against the Gram-positive bacteria therefore, it is combined with metronidazole to combat mixed infections [120,144]. Metronidazole has demonstrated efficacy against the obligate anaerobes originating from the necrotic pulp [153]. Minocycline (derivative of tetracycline) interfere with the bacterial protein synthesis [155]. The TAP in concentration of 1mg/mL (1:1:1) is widely used clinically for regenerative procedures and has shown promising outcomes in elimination of root canal microorganisms up to 99.99% [156] as well as promotion of revascularization in immature permanent tooth [157]. Due to acidic pH, TAP demineralized the dentin surface [148] that in turn favors the discharge of the entrapped cytoskeletons of the growth factors (such as TGF-β), cells differentiation and proliferation of dental pulp stem cells [158]. Moreover, demineralization enhances the surface roughness of the dentin that further facilitates the stem cells differentiation and attachment [159]. Meanwhile, TAP (1mg/mL or lower concentration) has no unfavorable effect on the stem cell viability [120,160]. However, concentrations of TAP higher than 1mg/mL have shown to be detrimental effects on dental stem cells [133]. This effect can be prevented by using low concentrations for regenerative endodontic [161]. Although tooth-staining due the presence of minocycline is common, this can be reduced by using low concentrations of TAP [162], and limiting TAP below the cementoenamel junction [163]. 

#### 4.3.3. Bioceramics

There is a wide range of bioceramics are used in dentistry including bioinert bioceramics (zirconia, filler part of restorative composites) and bioactive bioceramics (HA and CaP). The bioinert bioceramics are mainly used for restorative applications and discussed above in Section 3. The bioactive bioceramics are used in endodontics and can be categorized as bioresorbable (CaP bone substitutes) and non-bioresorbable (calcium silicate or hydraulic cements) [164,165]. Bioactivity induces a favorable response from host tissues such as deposition of HA layer when exposed to calcium and phosphate enriched tissue fluid [16,166]. Bioactive materials have good biocompatibility, osteoconductivity, osteoinductivity, and sealing ability. Gray mineral trioxide aggregate (GMTA) contains bismuth oxide, tricalcium and dicalcium silicates [167] and frequently used for endodontic applications including: vital pulp therapies, treatment of immature apices, sub-osseous perforation repair and as a root-end filling material [168]. ProRoot mineral trioxide aggregate (MTA) (GMTA; Dentsply Endodontics, Tulsa, OK, USA) is associated with discoloration of teeth when used in pulp-capping or pulpotomy procedures [169]. A white MTA ProRoot. MTA that lacks the tetracalcium aluminoferrite (white mineral trioxide aggregate (WMTA)); Dentsply Endodontics, Tulsa, OK, USA) was introduced in 2002 to overcome this concern [170]. Other drawbacks include cost, wash out during irrigation, delayed setting and difficult manipulation [171,172]. Despite, MTA rapidly became the gold standard for root-end restorations [173]

Improvement in endodontic materials continued by modifying different calcium silicate–based materials [174,175,176]. A relatively new materials, Biodentine (Septodont, Saint Maur des Fosse´s, France) has claimed to be a dentin replacement with similar indications as of MTA but with improved properties. The biodentine contain zirconia, tricalcium silicate and radio-opacifier [177] which interacts with the living cells and have proven good biocompatibility. Such interactions have been evaluated by several studies in vitro [178,179], ex vivo [180,181], in animals [182,183] or optimally in the clinics [184]. In regenerative endodontic protocols produced by the American Association of Endodontists [163] proposed to flood the root canals with blood by over-instrumentation (endo file, endo explorer) by passing through the apical foramen. Later, the blood clot may be replaced by either platelet-rich plasma [185] or platelet-rich fibrin [186]. The biodentine demonstrated the potential to overcome the major concerns of MTA (such as discoloration) and can be preferred in anterior teeth where the esthetic in the main focus [187]. 

## 5. Biomimetic Tissue-Engineering Aspects 

**Figure 4:**
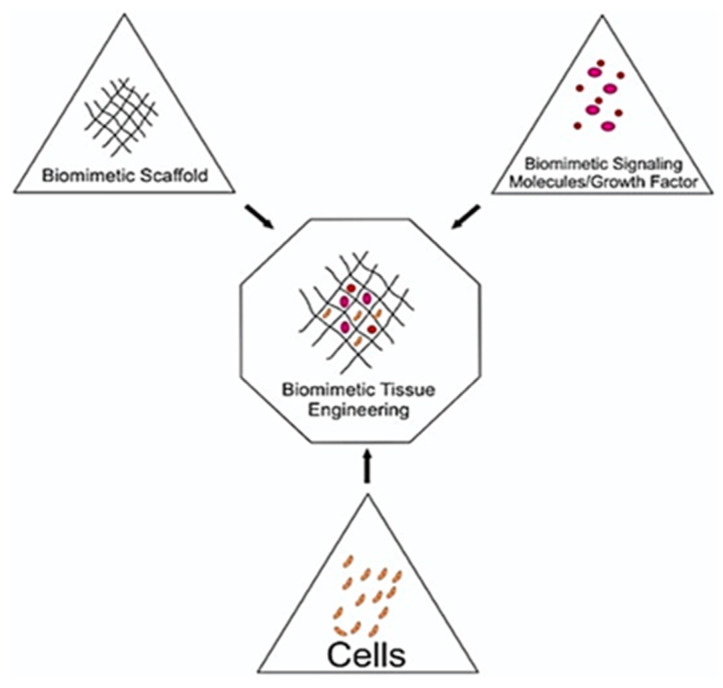
Fundamental components for biomimetic tissue engineering including biomimetic scaffold, signaling molecules and cells.

In 1993, Langer and Vacanti [188] proposed the concepts of tissue engineering in order to combat the donor scarcity to organ transplantation, immunosuppression issues and associated complications. Since then the field of tissue engineering has emerged enormously, thereby demonstrating its potential for regenerating almost every organ and tissue. Biomimetic tissue-engineering approaches target to mimic the intrinsic biological environment aiming to restore and improve the diseased or damaged tissues by tissues reconstruction or developing innate biological system. Biomimetic tissue engineering is a multi-disciplinary field and its goals can be achieved by merging knowledge of biology, chemistry, engineering, genetics and physics [189]. There are three fundamental principles applied in the field of tissue engineering [3,18]; (i) Implantation of biomimetic scaffolds that facilitates cells differentiation, proliferation and biosynthesis, (ii) The cellular adhesion with the surrounding tissues, through which new matrix can be synthesized and (iii) delivery of growth factors that support and indorse cells functions [190]. Figure 4 presents fundamental principles of biomimetic tissue engineering.

### 5.1. Desired Properties for Biomimetic Tissue-Engineering Scaffolds

**Figure 5:**
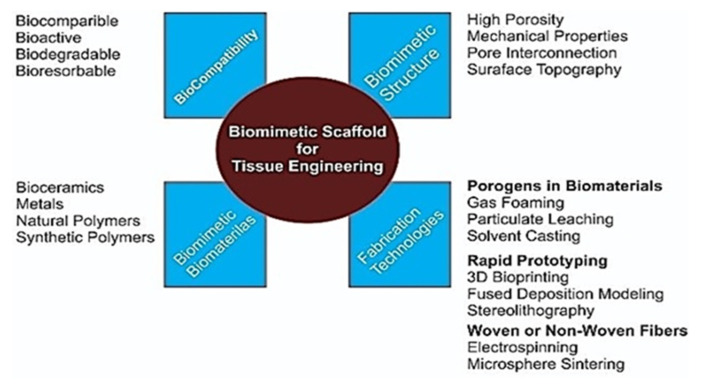
Key properties desired for a biomimetic scaffold for tissue engineering. (Reproduced from [193] with permission from the publisher).

Biomimetic scaffolds provide a three-dimensional (3D) microenvironment through a structural framework that can support cellular adhesion, vascularization, and organization. The cellular proliferation, differentiation and repair ultimately lead to the formulation of the desired or damage tissues [191]. The biomimetic scaffolds mimic extracellular matrix (ECM) architecture and composition through various mechanism, such as scaffold design, gene-based approaches, and cell-based therapies. To design scaffolds with particular desired characteristics, biomimetic, bio-resorbable and biodegradable materials have been explored extensively both experimentally and clinically across the globe [192]. To design an ideal biomimetic scaffold for the tissue regeneration is a complex and challenging task that shall fulfill a range of physical, mechanical, and biological properties (Figure 5). 

The biomimetic scaffold material and its breakdown products must be biocompatible and nontoxic to the target tissues. In terms of physical, and mechanical properties, the native tissues ECM should be considered to be a reference. For example, for bone regeneration, the physical and mechanical properties of scaffolds should mimic to that of bone tissues. Collagen is the major component of ECM organic matrix that controls architecture and cell behavior [193,194]. The diameter of nanofibers ranges from 50 to 500 nm [195,196]. High porosity and interconnected pores result in the facilitation in 3D regeneration of tissues, cell growth, differentiation and proliferation [197]. Mechanical properties of the biomimetic scaffold are another important feature that is crucial in biomimetic tissue engineering. The mechanical properties (compressive/tensile strength, elastic modulus, hardness, and fracture toughness) should be designed or modified in such a way that can they match with those found at the site of regeneration tissue [198,199,200,201]. Scaffolds fabrication using the functionally graded approaches mimic the morphology of natural tissues that can be advantageous [202]. Biomimetic scaffold involves immune-inert biomaterials model to regulate the immune system that applied immuno-modulatory concept in which decreased T and B lymphocytes and natural killer cell activity was mediated [203]. Another important property is the “bioactivity” of biomimetic scaffold. Bioactive glass scaffolds are fabricated using various techniques [166,204,205] and promote tissue neoformation in the host by appropriate cell integration differentiation and migration [206]. Bioresorbable and biodegradable features are also considered important for biomimetic scaffold formation. The scaffold material should have an adjustable and controllable biodegradation rate mimicking regeneration of the tissue [207]. Moreover, one of the critical factor for scaffold construction is excretion of these degradation products without interference with other organs of the body or causing any toxic effects [199]. Figure 5 represent the key desired properties of biomimetic scaffold for tissue engineering. 

### 5.2. Materials for Biomimetic Scaffold Fabrication

The biomimetic materials interact with the body biological processes during tissue regeneration and benefit individuals by replacing or repairing the damaged and pathological human tissues. In restorative dentistry, the biomimetic biomaterials are used for several tissue-engineering applications including regeneration of dentin, pulp, alveolar bone and cartilage, and restorative treatments [208]. A variety of materials including polymers, bioceramics, and metals have been explored for biomimetic tissue regeneration applications. 

#### 5.2.1. Polymers

Natural biomaterials represent potential viability and biocompatibility and further classified as polysaccharides (dextran, amylose, cellulose, chitin, and glycosaminoglycans), polynucleotides (RNA, DNA) and proteins (collagen, actin, fibrinogen, elastin, myosin, keratin, gelatin and silk) [209,210]. These materials are known as hydrophilic polymers based on self-assembly or cross-linking properties and mediate cellular degradation due to their inherent ability to interact with cells [211]. However, due to poor mechanical properties and stability, natural polymers are not preferred for the load-bearing applications [208]. Here is a brief review of the commonly used natural polymers in dentistry for biomimetic scaffold fabrication. 

Platelet-rich fibrin fabricates a biodegradable scaffold associated with the hepatocyte growth factor (HGF), connective tissue growth factor (CTGF), vascular endothelial growth factor (VEGF), and pro-collagen type I thrombospondin 1 (TSP-1). Moreover, it decreases expression of alkaline phosphatase (ALP) activity, osteocalcin (OCN) and bone sialoprotein (BSP) while up-regulating collagen-I and cementum-derived protein-23 [212]. The use of platelet-rich fibrin has been reported for various tissue regeneration applications including wound healing [213,214], periodontal defects [215,216,217], bone regeneration [218,219,220,221]. 

Collagen fulfils various requirements for biomimetic tissue-engineering scaffolds [222]. Its main benefit is enzymatic biodegradation and mechanical properties [223]. The properties of collagen can be altered by combination and cross-linking with inorganic compounds, e.g., HA [224]. In a study, mouse calvarial defect was implanted by collagen–HA scaffold derived from mesenchymal stem cells of mouse-bone marrow. The defect was healed after three weeks with biodegradation of collagen–HA scaffolds. Osteochondral defects can be successfully treated by human-derived bone MSCs along with collagen–HA scaffold [224]. The ECM mimics of collagen fabricated by electrospinning exhibited better osteoblastic differentiation on titanium [225]. Additionally, collagen-based biomimetic scaffolds have been explored after blending with other natural biomaterials including chitosan [226,227] and gelatin [228]. Gelatin is another natural, biocompatible, and biodegradable materials. Due to poor mechanical properties, it is mainly used for drug delivering and wound dressing applications [229]. For load-bearing applications of bone and cartilage repair, it is used in combination with other scaffold materials to adjust the mechanical properties [223]. Common scaffold materials blended with gelatin include ceramics [230,231], and chitosan [228,230,232,233].

Chitosan is a natural biomaterial that has a good biocompatibility and antibacterial activity [85,234,235,236] and has been used for several biomedical applications including drug delivery [237,238,239,240], dental tissues regeneration [227,241,242,243]. Chitosan scaffolds improved cell differentiation, attachment, and proliferation by providing a 3D matrix for dental pulp stem cells [228,244]. Researchers combined chitosan and other biomimetic biomaterials to synthesize composites and enhance the scaffold properties [245]. The combination of chitosan/carboxymethyl cellulose (CMC) with polyelectrolyte complex (PEC) up-regulated the expression of dentin sialophosphoprotein (DSPP) and osteonectin (ON) was developed by Chen et al. [245]. In addition, chitosan combined with tricalcium phosphate enhanced the in vivo vascular growth [246]. Biomimetic structure with layered macroscale with adaptable mechanical properties can be achieved by the combination of chitosan and type 1 collagen and seeded with HAT-7 dental epithelial cells and DSCs [247]. For bone regeneration applications, composite scaffolds of chitosan and hydroxyapatite has been reported [248,249]. Other biomaterials blended with chitosan included natural silk [250,251,252], collagen [253,254,255], alginate [256,257] and hyaluronic acid [258]. Hyaluronic acid in known for its excellent biocompatibility, biodegradability and viscoelasticity [223]. Its fast degradation rate as well as poor mechanical properties limits its use in the tissue-engineering applications. To improve the physical, cellular, and mechanical properties, hyaluronic acid is usually used as a composite in combination with other biomaterials such as chitosan [258] and alginate [259]. When hyaluronic acid is modified with hydrogel and arginine-glycine-aspartic acid (RGD) peptide, cell attachment, proliferative properties, cell interaction, and growth were greatly enhanced. Such combinations have been explored for pulp regeneration therapies and endodontics [260]. Good adhesion and proliferation of human adipose-derived MSCs was found when hyaluronic acid was modified with heparin- hyaluronic acid hydrogel for applications in biomimetic biomedicine [261].

Alginate can be used as a delivery system for tissue-engineering applications [223] that can be administrated via minimally invasive technique for bone and cartilage regeneration. However, its application is limited because of its weakened mechanical strength. Mechanical strength can be increased by calcium chloride as a cross-linking agent [262]. Natural silk is another natural polymeric biomaterial that is mainly obtained from silkworms [263]. The structural component of silkworm silk is silk fibroin that is highly hydrophobic and insoluble in majority of solvents including water [264]. Silk fibroin is a protein material composed more hydrophobic heavy chain and less hydrophobic light chain [265,266]. The amino acid composition of silk fibroin is mainly repeats of alanine, glycine, and serine forming mainly β-sheet secondary confirmation [267]. The silk fibroin primary and secondary conformation are responsible for the characteristic features such as crystallinity, hydrophobicity, and strength properties. For various biomimetic biomedical applications, silk fibroin demonstrated favorable properties such as excellent biocompatibility [268], biodegradability [269], mechanical properties [270] and capability to perform under variable condition of humidity and temperature [271]. Consequently, silk has been investigated for a range of biomedical applications including drug delivery [272,273,274] tissue-engineering scaffolds [275,276,277,278] and dentistry [279,280,281,282]. For biomimetic applications, silk biomaterials can be processed into a range of morphologies at the micron or even nanoscale such as coatings, films, foams, fibers, non-woven electrospun mats and hydrogels [264]. In addition, natural silk can be blended with other natural, synthetic and bioactive materials for producing composites of tailorable properties [251,283,284,285]. Therefore, natural silk-based biomaterials have a good potential for future tissue regenerative applications.

In addition to natural polymers, several synthetic polymers have also demonstrated improvement in cells attachment, potential to deliver soluble molecules, a controlled degradation rate, and capability to fabricate complex shapes [201,223,286,287]. Examples of synthetic polymers include polypropylene fumarate (PPF), polyanhydride, polyether ether ketone (PEEK), polycaprolactone (PCL), polylactic acid (PLA), and polyglycolic acid (PGA). Synthetic polymers have comparatively longer shelf life and can be produced in large uniform quantities cost effectively. Synthetic polymers have better mechanical and physical properties and can be used to replace both soft and hard tissues. In comparison with natural polymers, synthetic polymers have less ability to interact with cells [288] and biocompatibility issues. To overcome such disadvantages, the best solution is to fabricate composite scaffolds. In tissue-engineering application these biomimetic composite scaffold has improved biocompatibility and controlled degradation [208]. In bone-tissue engineering hydrogels is used as an important class of polymers which is also known as hydrophilic polymer networks that guide the growth of new tissues. Hydrogels can be both naturals (gelatin, alginate, and agarose) and synthetics (poly(vinylalcohol)-based). These materials allow cells to differentiate, adhere, and proliferate as they have ability to absorb water. Hydrogels are able to deliver bioactive molecules and mimic ECM topography and used in numerous tissue engineering [289].

#### 5.2.2. Bioceramics

CaP bioceramics are known for bioactivity and commonly used for fabricating synthetic bone grafts [166,204,290]. Among these are amorphous CaP (ACP), β-tricalcium phosphate (TCP), calcium-deficient hydroxyapatite, dicalcium phosphate dihydrate hydroxyapatite (HA, Ca_10_(PO_4_)_6_(OH)_2_), dicalcium phosphate anhydrous hydroxyapatite, monocalcium phosphate monohydrate, monocalcium phosphate anhydrous, tricalcium phosphate (TCP, Ca_3_(PO4)_2_) and octacalcium phosphate [291]. These bioceramics have been widely used in the regeneration of hard tissues due to their distinctive features such as biocompatibility, good bioactivity, osteoinductivity, and osteoconductivity. Recently, a bone defect due to ameloblastoma has been filled successfully with a nanocrystalline, ceramic HA enriched with magnesium [292]. In endodontics application, bioglasses (SiO_2_Na_2_O-CaO-P_2_O_5_) have been widely used because of high bioactivity. However, because of poor mechanical properties, high stability, and difficulty of shaping of these bioceramics limit their wide application in tissue engineering. 

#### 5.2.3. Metals

Metals are considered to be attractive materials for the construction of the 3D printing of biomimetic scaffolds for bone and tissue regeneration. Potential metals that are used in 3D printing of scaffolds are chromium, cobalt, stainless steel, titanium alloys and nitinol [293]. Recently, for biomimetic tissue applications degradable metallic biomaterials known as “biodegradable metals” (BMs) has been introduced. Zheng et al. referred BMs to gradual corrosion of metals that releases corrosion products which elicited appropriate host response as by in vivo. These products then completely dissolve with no implant residues which promote tissue healing [294]. Examples of BMs include Iron (Fe), calcium, magnesium (Mg) and zinc-based metals. Recently, BMs based on Mg [295] and Fe [293,296] have been used for scaffold fabrication but due to lack of data regarding biocompatibility and cell viability the potential of these materials is currently uncertain. Further research should be conducted using BMs to increase the availability of materials for biomimetic 3D printing scaffolds for bone regeneration.

Titanium (Ti) is a metal that is lightweight and used for replacement of bone because of its outstanding biocompatibility, corrosion-resistant, mechanical, and physical properties. Moreover, it is used as an implant material for effective osseointegration and bone ingrowth at the implant interface [297,298,299,300]. Due to the strong non-covalent binding abilities of graphene and graphene oxide (GO), it endorses osteogenesis and stem cell differentiation [301] To enhance the osteogenic potential of scaffolds graphene and its derivatives can be combined with other biomaterials [302,303]. Graphene is a planar, single layer of sp2 hybridized nonaromatic carbon atoms in hexagonal arrangement with a high electrical and thermal conductivity, surface area, and mechanical strength. It has immense applications in biomaterial devices drug delivery, structural materials and tissue engineering [304]. Because of the ordered structure it shows excellent antimicrobial activity, which is further enhanced in its oxidized form (GO). GO consists of functional groups that is oxygen and carbon atoms arranged in honeycomb structure making it a two-dimensional structure. GO has carbonyl and carboxyl groups on the edges whereas epoxide and hydroxyl groups on the basal plane [305]. The hydrophilicity and negative charge of GO stimulates its effective interaction with osteoblasts [306]. GO coating on titanium implant surface provides antibacterial activity and chemical functionalization that lead to oxidative and membrane stress in bacterial cells [307]. Antimicrobial properties are contributed by both oxidation and membrane stress. The steps included in its antimicrobial activity are on graphene-based materials cells will initially deposits followed by direct contact with sharp nanosheets which cause membrane stress on the cells and finally anion-independent superoxide oxidation will occur [308]. 

Kalisz et al. [309] investigated the corrosion properties of titanium alloy (Ti6Al4V) coated with graphene and compared with niobium pentoxide (Nb_2_O_5_). A considerable improvement in corrosion resistance behavior was observed in graphene coated titanium alloy than Nb_2_O_5_ coating [309]. Su et al. [310] successfully coated GO on Ti surfaces through dopamine and reported beneficial immunomodulatory effects and biocompatibility on osteogenesis in Ti-GO surfaces indicating that GO can be a possible coating material for the modification of implants and bone scaffolds [310]. In oral surgery, collagen membranes are used for treatment of bone defects. These collagen membrane does not permit the invasion of soft tissue into the growing bone. To improve the biocompatibility of bone and soft tissues a derivative of graphene that is graphene oxide is coated on the collagen membranes. Radunovic et al. [311] investigated the biocompatibility of collagen membranes coated with GO using DPSCs aiming to control inflammation event induction, ability to promote differentiation, and biomaterial cytotoxicity. The collagen membrane coated with GO demonstrated good biocompatibility and induced a faster DPSCs differentiation into bone cells. Therefore, GO is a potential substitute to conservative membranes thus ensuring more efficient bone formation and improving the clinical performance [311].

### 5.3. Methods of Processing 3D Biomimetic Scaffolds

Processing of biomimetic materials involves synthesis and design of new functional materials by modifying the structures, functions, processes, and biological products. Conventionally, these processes have reproduced by copying extracellular matrix functions and structures. In the last few years, several advancements have been reported for the construction of porous 3D biomimetic scaffolds. These contemporary scaffolds can be controlled at the nanoscale level for tissue regeneration. To explore new techniques and modifications, a plentiful research has been conducted focusing on manufacturing techniques for 3D scaffolds can be classified into three main types: 

1. Porogens in biomaterials; for example, particulate-leaching, solvent-casting, gas-foaming, phase separation, and freeze-drying [208]. Summary of these techniques along with their features are presented in Table 5.

2. Rapid prototyping techniques have been developed in the recent years including 3D Printing (3DP) Bioprinting (3D plotting or direct-writing), fused deposition modelling (FDM), selective laser sintering (SLS), and stereolithography (SL) [312,313,314,315] as presented in Table 6.

3. Woven or non-woven fibers scaffolds using electrospinning [316,317,318] and microsphere sintering [208]. The characteristics of various scaffolds fabricated using woven or non-woven techniques are presented in Table 7.

### 5.4. Dental Stem Cells Therapy for Biomimetic Tissue Regeneration

Dental stem cells (DSCs) are essentially required for regenerative tissue-engineering approaches [347,348,349]. Stem cells are undifferentiated, immature cells capable of cell differentiation and self-renewal [350]. These cells form ‘‘stem cell niche’’ and are reside in each tissue of specific areas. Adult stem cells residing in several mesenchymal tissues are referred as mesenchymal stem cells or multipotent mesenchymal stromal cells (MSCs). The MSCs are multipotent cells that can differentiate into multiple cells including adipocytes, chondrocytes, and osteocytes and various types of tissues, therefore attracted clinicians and researchers for regenerative applications [351]. Stem cells therapy is an advanced procedure for the treatment of degenerated tissues that can be used by administer cells of appropriate regenerative potential. There are various types and sources of post-natal dental stem cells. The characteristics of dental stem cells including biomimetic applications and associated cluster of differentiation (CD) are presented in Table 8. 

DSCs from dental pulp stem cells (DPSC), stem cell from human exfoliated deciduous teeth (SHED), and stem cells from the apical papilla (SCAP) are more commonly used [369]. Reprogramming of stem cells and somatic cell nuclear transfer are new stem cell technologies that are available to convert differentiated cells to the embryonic routes and overcoming the immune rejection that are very common problem occurred with embryonic cells [370]. To regenerate damaged oral structures, stem cell-based tissue-engineering approaches are promising [18]. However, to ensure safe and effective use of stem cell therapy, it is mandatory to understand the basic molecular mechanisms underlying stem cells fate [371].

### 5.5. Biological Cell Signaling Growth Factors for Biomimetic Tissue Engineering

In addition to the biomimetic scaffold and stem cells, biological cell signaling is an important component for biomimetic tissue engineering. Biological cell signaling is a complex system of communication that is responsible for the organization of the interactions within the cell and directs different cell activities [18,372]. Growth factors (GFs) or tissue inducing mediators play a major role in regulation of tissue differentiation. GFs are proteins that are formed by amino acid residues connected via polypeptides chains and act by binding to specific receptors and activating a series of signals during embryogenesis. For example. the cytoskeletons of GFs are embedded in the dentin matrix during dentinogenesis [373]. These growth factors once released again have the ability to make cellular responses [374]. These growth factors include TGF-β, platelet-derived growth factors (PDGFs), bone morphogenic proteins (BMPs), fibroblast growth factor (FGFs), and VEGFs [375]. Upon binding, specific cell fates occur after the triggering of a cascade. Certain type of cells is regulated by most GFs however, some act on multiple types of cells and have pleiotropic roles influencing numerous tissues. Different GF can have overlapping functions in many cases. Consequently, same kind of growth factors can be produced by different types of cells [376]. Additionally, these biological signals can maintain cellular development growth, proliferation and migration [377]. The secretion of GF from extracellular matrix occurs by enzymes and has a direct effect on restoration or tissue development. Several growth factors and their functions have been explored for tissue-engineering applications [378,379]. 

#### 5.5.1. Bone Morphogenetic Proteins 

Bone morphogenetic proteins play momentous role in formation of skeletal tissue during adulthood, embryogenesis, growth, and healing [380] as well as differentiation of various oral tissues including alveolar bone, ameloblasts, cementum and dentin during the tooth development [381]. Several studies have investigated various combinations of tissue-engineering scaffolds loaded with BMPs [249,256,382,383,384]. For dental tissue regeneration, the BMPs have been extensively explored [365,378,385]. BMPs signaling molecules are associated with the modulation and regulation of the functional and cytological differentiation of pulp cells into preodontoblasts and odontoblasts. BMPs (BMPs-2, 4 and 7) GFs induces bone regeneration at heterotopic sites and in vitro [380]. Bone morphogenetic protein-2 (BPM-2) has been reported to mediate the expression of sialophosphoprotein and odontoblast differentiation through NF-Y signaling [385]. When BMP-2 gene is directly delivered to tissue via an adenoviral vector healing of mandibular osseous defects was achieved [386]. 

The biological activity of BMP-7 was evaluated in two separate orthotopic regeneration models involving critical-sized calvarial defects and long bones and a periodontal alveolar defects [387]. The expression of several BMPs (BMPs 2, 3a, 4, 7 and 8) is observed that during fracture healing. The healing and regenerative capability of BMP-6 was assessed after creating periodontal defects in an animal study [388]. In this study, BMP-6 was delivered to the affected regions following onset of periodontal disease that was maintained for 8 weeks. The authors found that there increased growth of bone and periodontal ligament regeneration after BPMs-6 application confirming the significance of BMPs in regenerative periodontics [389].

#### 5.5.2. Vascular Endothelial Growth Factor 

VEGF promotes angiogenesis and vasculogenesis during tissue regeneration and persuades an angiogenic response in the severed pulp and assist in the revascularization process [390]. The calvarial defects treated using a combination of BMP-4 and VEGF demonstrated enhance bone regeneration compared to the transduced cells alone [391]. Several studies have reported the positive role of VEGF in dental pulp regeneration [390,392,393,394,395]. Yadlapati et al. reported that VEGF loaded biodegradable scaffolds enhanced cell viability, blood supply and survival of apical papilla stem cells [390]. In addition, using a combination of VEGF and BMP-2 promoted odontogenic and osteogenic differentiation of DPSCs [393]. Similarly, an overexpression of stromal cell-derived factor-1α (SDF-1α) and VEGF by DPSCs enhanced vascularization and dental pulp regeneration [394].

#### 5.5.3. Platelet-Derived Growth Factor 

PDGF also acts as angiogenic growth factor, tissue repair, proliferation, and osteogenesis. PDGF has the potential to regenerate hard and soft tissues and therefore have been explored for the periodontal [396,397,398], and bone regeneration [399,400]. However, there is a limitation of PDGF that at wound site their bioavailability and biological activity is transient because of the encoding of the PDGF receptor by growth arrest specific gene’. Recently researchers have developed PDGF-A gene transfer through adenovirus vector (Ad-PDGF-A) to overcome the limitation. This will release a sustained tyrosine phosphorylation which will extend the effect of PDGF on cell signaling that is critical for cells proliferation [401]. In addition, PDGF demonstrated proliferation of periodontal cells including fibroblast, cementoblasts and osteoblast during periodontal regeneration application [402].

#### 5.5.4. Fibroblast Growth Factor 

FGF is known to induce angiogenesis, chemotaxis and cell proliferation of periodontal ligament cells [403]. In terms of oral tissue regeneration, FGF is suitable for use in the periodontal tissues regeneration because in the periodontal ligament undifferentiated mesenchymal cells exist [404]. Nakahara et al. reported that after implantation controlled release of FGF not only maintained for at least 4 weeks but also involved in the wound healing of periodontal tissues [405]. 

#### 5.5.5. Transforming Growth Factor 

Transforming growth factor plays a major role of TGF-β in tissue homeostasis, osteo/chondrogenesis, tissue repair [376], embryonic development and numerous pathological conditions [406,407]. The TGF-β regulate several genes associated with differentiation, growth and wound healing through the serine/threonine kinase receptors. In addition, TGF-β signaling regulates cell membrane by a proteolytic processing and release from the extra cellular matrix through its bioavailability [408]. TGF-β inhibits proliferation of macrophages and neutrophils, suppress T cell maturation and chemotactic migration thereby function as effective regulator of adaptive and innate immunity [409]. Moreover, it was found that when growth factors such as TGF-β and BMPs were used in combination, induces enhanced osteo-inductive activity and regeneration of bone using these proteins alone. Tachi K, at el [410] investigated a combination of BMPs and TGF loaded to collagen scaffolds and reported improved bone healing and regeneration with improved osteo-inductive activity the bone regeneration [410]. Studies reported that TGF-β may be released following chemical treatment of the dentin such as washing with EDTA [159]. Moreover, it has been shown that extreme pH levels (1.5 or 12) help to activate TGF-β [159], making it the fundamental molecule for pulp revascularization. 

This concept of controlling release profile has been applied to the biomimetic mineralization process. Biomimetic coprecipitation technique showed gradually released of BMP-2 dose where as in superficially adsorbed technique burst release of BMP-2 was observed [411]. The efficacy and the delivery mode of BMP-2 were tested using Ti-alloy (Ti6A14V) discs implanted in the dorsal region of rats subcutaneously for up to 5 weeks. The results showed that the groups which were provided with sustained delivery of BMP-2 demonstrated improved density and volume of the regenerated bone [412,413]. All these biological signaling molecules and growth factors have the potential to modulate the regeneration and repair process [414]. These growth factors have limited cost-effectiveness when injected into the target site. In addition to the short half-life the cells are also sensitive to the concentration of these biological signaling molecules the effective application of growth factors in biomimetic tissue regeneration mainly dependent on delivery technologies. These delivery technologies directly affect the time for which the growth factors remain active in target tissue. This situation has provoked the use of systems capable of retaining these biomimetic growth factors for prolonged period. The purpose of these delivery technologies is to control the delivery of growth factor to encourage therapeutic action or tissue formation. Nevertheless, providing precise delivery of these biological molecules is a challenging task in the field of biomimetic tissue engineering. Ongoing research discovered a wide range of techniques and strategies to control the release kinetics of biological signaling molecules. State-of-the-art delivery techniques include scaffold immobilization by physical or chemical encapsulation, microencapsulation, microspheres, and triggered delivery. Further investigations has been conducted on the robust delivery systems which involve more than one approaches [376].

**Figure 6:**
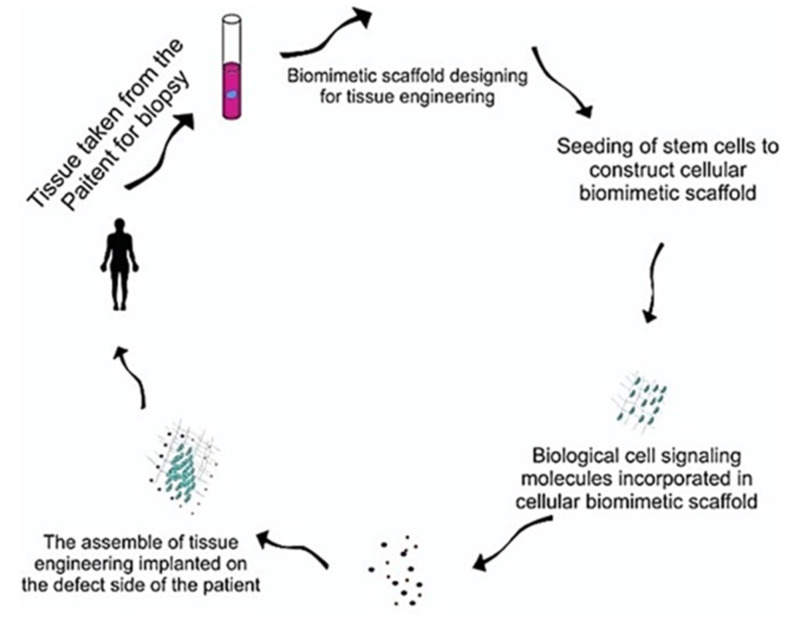
Diagrammatic representation of basic components and steps for the biomimetic tissue-engineering concept for dental tissue regeneration. (Reproduced from [416] with permission from the publisher).

A simple schematic concept of biomimetic tissue engineering using three basic components is shown in Figure 6. The delivery of these proteins within the minerals tissue regeneration can be by two mechanisms: First, proteins can adsorb on the mineral surface through electrostatic interaction between proteins and mineral apatite. This is known as surface adsorption method. Secondly, by coprecipitation in simulated body fluids solution which is known as coprecipitation method. the release kinetics of the incorporated proteins through these processes are different which affect the properties of the mineral structure. More controlled and sustained release profile occur from coprecipitated proteins whereas the surface-adsorbed proteins demonstrate a burst release profile [411,412,415]. It is understood from the previous studies that different growth factors result in different morphological characteristics. In, future, there are chances to option for engineering dentin morphological characteristics by selection of specific biomolecules according to the required tissue regeneration [374]. Although the tissue-engineering approaches demonstrated promising outcomes, there are still very limited applications in clinical scenarios. The regeneration of oral and dental tissues mimicking natural tissues is challenging due to their complex structural and functional nature. The fabrication of an ideal scaffold with controlled materials properties such as porosity, surface to volume ratio, and pore distribution is crucial however not straight forward. Additional challenges included the appropriate organization of the events that occur at healing site so that it will results in cell promotion and growth. Further research exploring signaling molecules and new biomaterials are therefore of great interest and essentially required for the progress of oral tissue engineering and regeneration.

## 6. Conclusions and Future Trends

In order to develop biomimetic restorative biomaterials, plenty of research has been conducted either modifying the existing materials or developing new materials. A variety of processing technologies including nanotechnology, fabrication methods, and functionalization of biomaterials has been explored. In the past decade, biomimetic restorative materials demonstrated considerable advancements in their properties simulating that of natural tissues. However, due to the complex structural and functional nature of dental tissues, the development of biomimetic restorative materials is still at the preliminary stage. Similarly, biomimetic tissue engineering has experienced exponential growth from developing theoretical phase to a multi-faceted fast-emerging field in recent decades; however, translating such developments to practical and clinical applications requires further research. 

Considering the major challenges faced by researchers and clinicians, perhaps it may take a more than a decade for biomimetic materials to be implemented on a bigger scale to treat dental lesions. New alternative treatment modalities are likely to be available for clinical applications after innovative discoveries in genetics, molecular biology, cell biology, and materials science. Through these treatment modalities, regeneration of dentin, enamel, pulp, restorative procedures, and management of soft tissues of periodontium can possibly be carried out. In the near forthcoming years the reinforcement and completion of the tooth structure by biological regeneration will be evident through these modalities. The development and translation of smart biomimetically driven dental restoratives from lab to clinical dentistry also have a tremendous potential. But there are still numerous challenges and limitations for clinical applications and predictable outcomes due to complex natural tooth structure. However, biological and biochemical mechanisms related to biomineralization would require further expansion of knowledge. The possibility of using novel biomaterials with innovative biomimetic cell-free templates, intrinsic disordered protein, and efficient peptide-based remineralization strategies may have conceivable perspective. Moreover, the role of various biomimetic agents and molecules involved in the regeneration of tooth tissue would require further study. Nonetheless, plentiful interdisciplinary research is under way to develop biomimetic materials. We hope for the availability of completely regenerated dental tissues (enamel, dentin, pulp, and cementum) with biological, mechanical, and mineralized nano-structural properties mimicking that of natural tooth tissues. 

## Figures and Tables

**Table 1:** Key functions of dentin–pulp complex [25].

Formative (Developmental)	Odontoblasts Produce Primary and Secondary Dentin
Nutritive	Supply of nutrition to odontoblasts processes through the blood supply (mineral ions, proteins, and water to dentin)
Sensory (protective)	Pulpal nerve fibers mediate the sensation of pain (motor and sensory nerve fibers initiate reflexes)
Defensive (reparative)	The DPC: Response to pathologic challenges

**Table 2:** Modulus of elasticity of tooth-colored restorative materials and tooth hard tissues.

Restorative Materials	Test	Elastic Modulus (GPa)	References
Tooth Hard Tissues			
Tooth enamel	Nanoindentation	72.0–125.0	[51]
		80.9 ± 6.6	[50]
Tooth dentin	Nanoindentation	14.0–38.0	[51]
		20.5 ± 2.0	[50]
Resin-Based Dental Composites (RDCs)			
Z100 (Micro-hybrid RDC), (3M ESPE, USA)	Three-point bending	18.3 ± 1.2	[69]
		11.3 ± 0.5	[39]
Z250 (Micro-hybrid RDC), 3M ESPE, USA	Three-point bending	16.7 ± 0.8	[69]
		6.9 ± 0.6	[39]
Flitek Supreme (Nanofilled RDC), 3M ESPE, USA	Three-point bending	13.7 ± 0.6	[69]
		9.4 ± 0.7	[35]
Tetric Ceram (Hybrid RDC), Vivedent Schaan Liechtenstein	Three-point bending	6.9 ± 0.5	[70]
		9.4 ± 0.9	[71]
Clearfil PhotoPost (Hybrid RDC) Kuraray, Osaka, Japan	Three-point bending	18.0 ± 1.2	[70]
Point 4 (Flowable RDC), Kerr, Orange CA, USA	Compression	3.5 ± 0.87	[72]
Grandio (Hybrid Paste RDC), Voco (Cuxhaven, Germany)	Three-point bending	15.3	[73]
Glass–Ionomer Cements (GIC)			
Riva Light (RMGIC) SDI, Victoria, Australia	Three-point bending	2.1 ± 0.4	[74]
Aqua ionofil U (conventional GIC, Voco, Cuxhaven Germany	Dynamic mechanical analysis (DMA)	1.8 ± 0.01	[75]
Fuji II LC (FL) (RMGIC)G. C. Belgium N.V.	DMA	5.8 ± 0.02	[75]
Riva Self-Cure (Glass–ionomer cement) SDI Limited, Victoria, Australia	Three-point bending	6.3 ± 1.3	[76]
Fujji IX, GC, Australlia	Three-point bending	7.8	[77]
Ionofil Molar Glass–Ionomer Cement, Voco, Cuxhaven, Germany	Indentation	12.3 ± 2.1	[78]
Dental Ceramics			
e.max Press (Lithium-disilicate-based glass-ceramic). Ivoclar Vivadent, Schaan, Liechtenstein)	Ultrasonic pulse-echo method	82.3	[41]
PM9 Vita (Feldspathic-based ceramic), (VITA Zahnfabrik, Bad Säckingen, Germany)	Ultrasonic pulse-echo method	44.4	[41]
IPS e.max^®^ Press glass-ceramic material Ivoclar Vivadent AG, Schaan, Liechtenstein	Deflection	95.0	[79]
Vita In-Ceram alumina core. Vita Zahnfabrik	Three-point bending	271.3	[80]
#Vita Suprinity (Zirconia reinforced lithium silicate glass-ceramic) and Vita Zahnfabrick, Bad Säckingen, Germany	Three-point bending	70.4 ± 1.9	[81]
IPS e.max (Lithium disilicate glass-ceramic) Ivoclar Vivadent, Schaan, Lichtenstein	Three-point bending	60.6 ± 1.6	[81]

**Table 3:** Surface hardness of tooth-colored restorative materials and tooth hard tissues.

Restorative Materials	Test	Surface Hardness (SH)	References
Tooth Hard Tissues			
Tooth enamel	Nanoindentation	2.2–7.2 GPa	[51]
		4.9 ± 0.4 GPa	[50]
Tooth dentin	Nanoindentation	0.7–0.9 GPa	[51]
		0.9 ± 0.1 GPa	[50]
Resin-Based Dental Composites (RDCs)			
X-tra fil (bulk-fill) Micro-hybrid RBC, Voco, Guxhaven, Germany	Vickers	75.8 ± 7.0 VHN	[43]
		70.9 VHN	[73]
QuiXfil (bulk-fill) Micro-hybrid RBC, Densply, Konstanz, Germany	Vickers	64.1 ± 6.2 VHN	[43]
Grandio Nano-hybrid RBC, Voco, Guxhaven, Germany	Vickers	120.8 VHN	[73]
		92.6 ± 6.1 VHN	[43]
Z100 Micro-hybrid RBC, 3 M ESPE, USA	Knoop	74.1 ± 9.0 KHN	[82]
		120.8 ± 15.1 KHN	[83]
Filtek Supreme Nanofilled RBC, 3 M ESPE, USA	Knoop	58.4 ± 3.6 KHN	[82]
		42.8 ± 6.2 KHN	[82]
Filtek Z250, Micro-hybrid RBC, 3M ESPE, USA	Vickers	72.0 VHN	[84]
		82.0 ± 4.0 VHN	[85]
Glass–Ionomer Cements (GICs)			
Fuji IX, GIC, GC Corporation, Tokyo, Japan	Vickers	26.4 ± 4.4 VHN	[46]
	Knoop	68.7 ± 10.9 KHN	[63]
Ionofil Molar Glass–Ionomer Cement, Voco, Cuxhaven, Germany	Vickers	74.3 ± 6.7 VHN	[46,85]
		57.4 ± 15.2 VHN	[78]
KetacTM Molar Easy Mix, GIC, 3M-ESPE, Saint Paul, USA	Knoop	77.5 ± 37.7 VHN	[86]
Equia Forte Glass–Ionomer Cement GC, Tokyo, Japan	Vickers	120.1 ± 10 VHN	[87]
Vitromolar GIC, DFL Ind’ustria e Com’ercio Ltd.a (RJ, Brazil)	Vickers	40.9 ± 4.3 VHN	[88]
		40.6 ± 0.8 VHN	[88]
Dental Ceramics			
Alumina Ceramic (Fabricated using slip casting technique)	Vickers	1679 HV	[89]
Alumina/Zirconia Ceramics, (Fabricated using the slip casting technique)		1447 HV	[89]
Duceram love Dental Ceramics, (Degu Dent GmbH, Denstply, Germany)		6.1 ± 0.7 GPa	[44]
IPS e.max ceram Dental Ceramics, Ivoclar- Vivadent AG, Germany		6.1 ± 0.3 GPa	[44]
Feldspathic ceramic Block, Vita Zahnfabrik, Germany		502.4 ± 2.3 kg/mm^2^	[90]
Vita VMK 68 Leucite Dental Ceramics, Vita Zahnfabrik, Germany		6.9 ± 0.1 GPa	[91]

**Table 4:** Surface hardness of tooth-colored restorative materials and tooth hard tissues.

Irrigant	Main Outcome	Reference
CHX (2%)	No survival of SCAPs Toxicity to SCAPs	[127] [130]
NaOCl (6%)	Survival of SCAPs (combined to the 17% EDTA) Reduced survival of SCAPs	[127] [128]
NaOCl (1.5%)	Survival of SCAPs	[128]
EDTA (17%)	Survival of SCAPs	[127,128]

**Table 5:** Summary of the materials processed, advantages and disadvantages of porogens in biomaterials for biomimetic scaffold production.

Technique	Materials Processed	Advantages	Disadvantages
Thermally induced phase separation (TIPS) [319,320,321]	poly(L-lactic acid)-based scaffolds	Processing flexibility. Produce 3D scaffolds Enhanced osteogenic differentiation. Induction of defect healing.	Uncontrolled pore distribution and size. Limited to a few polymers. Lack of control over 3D shapes
Supercritical fluid-gassing [322,323]	poly(DL-lactic acid-coglycolic acid) (PLGA), Poly (DL-lactic acid) (PDLLA)	Preparation of an exact porous copy. Organic solvent is not required.	Decrease pore size. Fragile scaffold. Form nonporous layer. Take hours to complete.
Self-assembling [324,325]	Hydrogel scaffold and peptide-amphiphile (PA)	To engineer soft and hard mineralized matrices for dental/pulp tissue regeneration.	Inability to controlled macro-sized pores. Limited formation of mechanically stable 3D geometry.
Emulsion freeze-drying method/ lyophilization [289,326,327]	Natural and synthetic polymers	Highly porous scaffolds. Large surface areas. Superior mechanical properties. High temperatures can be avoided. Good biocompatibility. Extensive osteoconductivity.	Inadequate control of scaffold pore size, network and architecture. Lengthy procedures. High consumption of energy. Use of cytotoxic solvents. Formation of irregular, small size pores (15–35 μm).
Gas-foaming process [319,328,329]	PLGA	Highly porous. Organic, cytotoxic solvents are not required. Inert gas-foaming agents.	Technique cannot be used for hydrophilic and glassy polymers. Use of excessive heat. Close, non-interconnected pore structures.
Solvent-casting and particulate-leaching [319,327,330]	PGA	Most common and easy method. Sustainable equipment costs Pore size and porosity can be controlled. High porosity and interconnected pores. Capable of healing critical bone defects in rat femoral medial epicondyles.	Harmful residual solvent. Decrease in the activity of bio-inductive molecules. Impossibility of adding pharmacological agents. Process can only form simple shape scaffolds

**Table 6:** Summary of the materials processed, advantages and disadvantages of woven or non-woven fibers technique for biomimetic scaffold production.

Technique	Materials Processed	Advantages	Disadvantages
Electrospinning [331,332,333]	Natural polymers: collagen, silk fibroin, and fibrinogen, chitosan, gelatin Synthetic polymers: PGA, PLLA, PLGA, and PCL)	Accurate porosity and morphology. Fibers within nanometer range. Strong ability to induce osteogenic differentiation. Small pore size mimics the ECM, density and high surface area.	Require organic solvents. Difficult to create large 3D scaffold clinically.
Microsphere sintering [334,335,336]	Synthetic polymers: PLGA	Improved cellular attachment and proliferation. Excellent mechanical properties.	CO_2_ was used that creates a closed-pore structure.

**Table 7:** Summary of the materials processed, advantages and disadvantages of rapid prototyping techniques for biomimetic scaffold production.

Technique	Materials Processed	Advantages	Disadvantages
Stereolithography [330,337,338]	Synthetic polymers: PEG, PEGDA, PPF, PCL, PDLLA	Highly accurate scaffold Greatly improved adhesion, proliferation, and osteochondral differentiation. Easy removal of photopolymer by heating.	Skin irritation and cytotoxicity Photo-polymerization of materials. Expensive materials and equipment
Fused deposition modelling (FDM) [330,339,340]	Synthetic polymers: PCL, PLGA, PC, PPSF, PEI, PVA, ABSP400	High porosity and controlled pore size with a complete interconnectivity Good mechanical strength No need for toxic solvent. Flexibility in material processing. Controlled porosity and size of pores.	High processing temperature. Limited material range. Inconsistent pores. Application to biodegradable polymers may be limited.
Selective laser sintering (SLS) [330,341,342,343,344]	Synthetic polymers: PEEK, PCL, poly(lactic acid) Ceramics: HA, TCP	High compressive strengths. Solvent-free. Complex structure. Can control pores size and porosity.	Needs powder materials that should withstand laser heat. During sintering process materials should resist shrinkage of the scaffold Pre- and post-heating treatments of the powdered material. Thermally stable polymers can be used. Limited/small pore size
Three-dimensional bioprinting [330,345,346]	Ceramics Polymers Hydrogel Metals	Easy process. High porosity with a controllable pore size and complete interconnectivity. Enhance cell attachment and regeneration. Capable of creating customized scaffolds that precisely fit the patient’s need.	Lack of mechanical strength. Lack of integrity. Use of toxic organic solvent.

PEGDA: poly(ethylene glycol)diacrylate, PEG: polyethylenglycol, PCL: polycaprolactone, PPF: polypropylene fumarate, PVA: polyvinyl alcohol, ABSP400: acrylonitrile-butadiene-styrene, PDLLA: poly D,L-lactide, TCP: tricalcium phosphate, HA: hydroxyapatite, polycarbonate polyetherimide (PEI), and polyphenylsulfone (PPSF) polyetheretherketone PEEK, polycaprolactone, PCL.

**Table 8:** Summary of human-derived dental tissues mesenchymal stromal cells (MSCs): isolation, multi-potentially, source, and cd antigen expression and their clinical application in biomimetic tissue engineering.

Cell Type	Multi-Potentially	Source	Biomimetic Applications	CD * Antigen Expression	References
Dental pulp stem cells (DPSC)	Adipogenic Chondrogenic Myogenic Neurogenic Osteogenic Odontoblast	Pulp of natal, supernumerary, and impacted third molars. Inflamed pulp. Cryopreserved healthy molars and premolars. Diseased but vital teeth.	Regenerative endodontics Bone regeneration	Positive: CD9, CD10, CD13, CD29, CD44, CD49d, CD59, CD73, CD90, CD105, CD106, CD146, CD166 Negative: CD14, CD31, CD34, CD45, CD117, CD133	[352,353,354,355,356,357]
Stem cells from human exfoliated deciduous teeth (SHED)	Adipogenic Chondrogenic Dentinogenic Myogenic Neurogenic Osteo-inductive Odontoblast	Remnant pulp of exfoliated deciduous teeth.	Regenerative endodontics and bone regeneration	Positive: CD13, CD44, CD73, CD90, CD105, CD146 Negative: CD14, CD19, CD34, CD43, CD45	[358,359,360,361]
Periodontal ligament stem cells (PDLSC)	Adipogenic Chondrogenic Myogenic Neurogenic Osteogenic Cementogenic	Periodontal ligament (PDL) of healthy permanent teeth. Inflamed regenerating PDL from intrabony defects	Periodontal regeneration Bone regeneration	Positive: CD9, CD10, CD13, CD29, CD44, CD49d, CD59, CD73, CD90, CD105 CD106, CD146, CD166 Negative: CD31, CD34, CD45	[353,362,363,364]
Dental follicle stem cells (DFSC)	Osteogenic, adipogenic, and periodontium-like tissues differentiation capacity Osteoblasts, Chondrocytes, Adipocytes	Normal human impacted third molars	Periodontal Regeneration Bone regeneration	Positive: CD9, CD10, CD13, CD29, CD44, CD49d, CD59, CD73, CD90, CD105, CD106, CD166 Negative: CD31, CD34, CD45, CD133	[353,365,366,367]
Stem cells from apical papilla (SCAP)	Adipogenic Chondrogenic Dentinogenic Myogenic Neurogenic Odontoblast Cementoblast-like cells	Immature roots of normal human impacted third molars	Regenerative endodontics Bone regeneration	Positive: CD49d, CD51/61, CD56, CD73, CD90, CD105, CD106, CD146, CD166 Negative: CD14, CD18, CD34, CD45,	[352,358,368]

* Cluster of differentiation (CD).
